# A longitudinal study examining emotional, behavioural and attachment disorder symptoms of care-experienced young children: the role of early adversity and age at entry to care

**DOI:** 10.1007/s00787-026-03014-6

**Published:** 2026-04-02

**Authors:** Jessica Oldridge, Karen Crawford, Enrico Venturini, Helen Minnis, Jala Rizeq

**Affiliations:** 1 School of Health and Wellbeing, University of Glasgow, Glasgow, UK; 2 Gillberg Neuropsychiatry Centre, University of Gothenburg, Gothenburg, Sweden

**Keywords:** Foster Care, Mental Health, Attachment, Adverse Childhood Experiences

## Abstract

Care-experienced children are known to have histories of adverse childhood experiences (ACEs). Exposure to adversity is a well known risk factor for negative outcomes across the lifespan. However, very little is known about the adversity histories of young children in care, and how this may affect their emotional, behavioural, and attachment disorder outcomes over time. Data from 378 young children in foster care, aged 0–5 years old, as part of the Best Services Trial (BeST^?^) were used. Adversity experiences were identified and coded by reviewers from social care records. Age at entry to care was also recorded. Outcome variables used in this study included emotional and behavioural difficulties and attachment disorder symptoms measured at two timepoints (a few weeks after entering care, and 2.5 years later). The number of ACEs in this sample was high, with around two thirds of children having experienced 4 or more. The older the age at which a child entered foster care was strongly related to higher number of ACEs, and increased severity of maltreatment, experienced. An increase in emotional and behavioural difficulties after 2.5 years in care was significantly uniquely predicted by the number of pre-care ACEs a child experienced. Attachment disorder symptoms were significantly lower at 2.5 years in care as compared to levels at entry to care. These findings indicate that prevention and early identification of risk in vulnerable families is paramount. Additionally, tailored interventions for the emotional and behavioural wellbeing of young children in care is essential.

## Introduction

Early life experiences are important in shaping the development and psychological wellbeing of young children [1], making this area of work a global research priority, particularly within the context of early adversity [2] and its impacts on lifespan health and wellbeing. Adverse childhood experiences (ACEs) are highly stressful events, and include maltreatment (emotional, physical or sexual abuse, or neglect), domestic violence and parental mental health or substance misuse problems [3]. Around 90% of care-experienced populations will have experienced at least one ACE [4, 5]. Data from young (under 5 years) care-experienced children are scarce, but initial research suggests that pre-care adversity for younger children may be characteristically different from those who are older, with experiences of neglect being prominent in this age group [6].

Early exposure to abuse and neglect by caregivers and disruption in care can contribute to a sense of insecurity and lack of safety in child-caregiver relationships and result in significant developmental, emotional, behavioural and relational difficulties (for meta-analyses see [7, 8]). With repeated exposure to maltreatment, some children may go on to develop attachment disorders (i.e. Reactive Attachment Disorder (RAD) and Disinhibited Social Engagement Disorder (DSED), whereby the patterns of behaviour become pervasive across various relationships [9, 10]. Attachment disorders are directly related to exposure to maltreatment and difficulties arise before age five [11]. Children with RAD find it difficult to form, seek and accept closeness from others, showing heightened behavioural outbursts, emotional dysregulation, and fearfulness [11]. On the other hand, a child with DSED will demonstrate indiscriminate behaviour towards adults, such as willingness to leave caregivers for other adults or engage in familiar behaviour (e.g. hugging) with strangers [11]. Attachment disorders range between 1 and 2% in the general population [12]. Exposure to ACEs generally, beyond maltreatment, has also been linked to higher risk of attachment disorders and emotional and behavioural problems in children [13–15].

In care-experienced populations, there are high rates of mental health problems including attachment disorder symptoms. In a meta-analysis of studies looking at pre-school children in foster care, Vasileva & Petermann (2018) found much higher rates of emotional and behavioural difficulties than would be expected in normative samples. Often these presentations can be chronic, with longitudinal studies demonstrating symptomatology persists over years despite removal from adverse environments [16–19]. RAD and DSED symptoms are also heightened in care-experienced children. One longitudinal study recruiting 4- to 8-year-olds found that those in residential and adoptive placements had higher symptoms of both attachment disorders than community controls, although symptoms in those who were adopted did decrease over time spent in stable placements [9].

Another important factor implicated in care-experienced children’s mental health and wellbeing is age at entry to care, whereby earlier placement can mitigate exposure to adversity and offer opportunities to establish healthy relationships [20]. In a small sample of 43 pre-school children in care, Hillen and Gafson, [21] showed that entering care after 6 months of age was associated with more emotional, behavioural and attachment disorder symptoms than those placed in care earlier. Similarly, in a longitudinal study of adoptees with pre-adoption history of institutional deprivation, those who experienced over 6 months of early institutional deprivation showed significant impairment in a range of outcomes including DSED symptoms that persisted across time [19]. Therefore, age at entry to care and/or duration of adversity are important considerations when examining outcomes in the care-experienced population.

The overall aim of this study was to comprehensively characterize the type and severity of adversity histories experienced by young children prior to entry to care and explore their role in shaping mental health outcomes. Specifically, we aimed to address the following research questions:

1. What are the characteristics of adverse experiences in young children in foster care, based on social care records?
2. To what extent are adversity and age at entry to care related to the emotional, behavioural and attachment disorder symptoms in in young children in foster care cross-sectionally and over time?

## Method

### Participants and procedure

This study utilises quantitative longitudinal data from a randomised controlled trial named the Best Services Trial (BeST^?^), conducted between 2011 and 2022 [22]. BeST^?^ compares an infant mental health service intervention (using the New Orleans Intervention Model) with social work care-as-usual for pre-school children entering the foster care system. BeST^?^ recruited participants from Greater Glasgow and Clyde and several South London boroughs. Due to the availability of specific variables required for the current analysis (i.e., access to social care records for ACEs), participants from the London sites (*n* = 110) were not included in the current study, leaving 378 participants from Glasgow. Participants’ quantitative outcome measures were taken at 3 timepoints over the duration of the trial; Time 1 (T1) at a few weeks post entry to foster care, Time 2 (T2) at 15 months post accommodation, and Time 3 (T3) was around two and a half years later. While data were collected over three timepoints, Time 2 (T2) data were excluded from the current analysis due to disruption in data collection caused by the COVID-19 pandemic.

All families with children under 5 years of age entering foster care in Greater Glasgow and Clyde since 2017 were offered to take part in the study, with a successful recruitment rate of around 60% and nearly 80% retained over 2.5 years [23]. The only exclusion criteria for the overarching study were if biological parents were unable to engage in the trial due to death or long-term imprisonment. No further exclusions were applied in the current study.

The current study included data from 378 participants (young children and their carers) recruited to BeST^?^. Of those, 193 received the New Orleans Intervention Model and 185 received social work care as usual. Of the total sample, 52.9% of child participants were male. The average age of entry to care, and to the trial, was just over 2 years (M = 2.17, SD = 1.61, Range = 0.04–5.58 years). Most children in the sample were living in the most deprived areas of Scotland, with 69.05% living in the first decile of the Scottish Index of Multiple Deprivation (SIMD). No children lived in the three least deprived deciles and three participants did not have recorded SIMD.

## Measures

### Age at entry to care

This is the age in months of child participants when they entered the trial (T1), and therefore the age they were when they entered foster care.

### Deprivation level

This was measured using the Scottish Index of Multiple Deprivation [24]. This tool, created by Scottish Government, provides a single deprivation index score based upon postcode. It is derived from seven deprivation indicators including income, health, crime and housing provision. These data were based upon the biological family’s postcode.

### Adversity and maltreatment history

ACEs were coded using the Adverse Childhood Events Questionnaire (ACE-Q; [3]). This is a 10-item questionnaire completed by the research team with the child’s family, carers and records. Each item requires a yes for present or no for absent response. The 10 types of ACEs included: Emotional Abuse, Physical Abuse, Sexual abuse, Emotional Neglect, Physical Neglect, Parental Separation, Domestic Violence, Substance Abuse, Household Member Mental Illness and Household Member Incarcerated. A cumulative ACEs score is calculated based on the sum of all 10 responses, with a possible range of scores from 0 to 10. Experiencing four or more ACEs is considered a ‘high’ level of ACEs related to significant levels of difficulty [3, 25].

Exposure to maltreatment was coded by research team members based upon children and families’ social work records. The system used was the Maltreatment Classification System (MCS) [26], with additional content added from the Modified Maltreatment Classification System (MMCS; English & the LONGSCAN Investigators, 1997). This system categorises maltreatment type; Physical Abuse, Sexual Abuse, Emotional Maltreatment, Physical Neglect – Failure to Provide, Physical Neglect – Lack of Supervision, Moral-Legal/Educational Maltreatment. The MCS allows for coding of severity of maltreatment. Total maltreatment severity was the primary outcome and was calculated using the method by Litrownik et al. [27], whereby the ratings for the neglect and abuse maltreatment subtypes are summed. For total maltreatment severity, the maximum score is 54, with higher scores indicative of higher severity. This coding system is well used in maltreatment research [28].

All raters who completed the ACE-Q and MCS received comprehensive training and supervision throughout the process. Initial training was delivered via presentation, guidance notes with proformas and worked examples. New raters trained together and rated together in pairs for first several ratings until they were confident that they were largely rating in agreement. Then raters moved on to independently review and rate. Regular inter-rater sessions were held during which tricky cases were brought to rate in conference with an expert rater and where small numbers of randomly selected cases were chosen to be rerated to consider agreement with existing rating. Of the total ACE-Q, 22.12% were double rated and of the MCS, 12.95% were double rated. During the check in sessions and conferences, no major issues with agreement levels were flagged.

### Symptoms of attachment disorder

These were measured by The Disturbances of Attachment Interview (DAI) [29]. This caregiver interview includes 12 items exploring clinical symptoms of Reactive Attachment Disorder (RAD) and Disinhibited Social Engagement Disorder (DSED).

This version of the DAI was utilised as the revised version [30] was not available at the time of trial commencement. The new version is adapted to align with the introduction of the DSM-V criteria for attachment disorders. The key difference between DSM-IV and current criteria is that it DSED and RAD are conceptualised as distinct disorders, whereas previously they were defined as disinhibited (now conceptualised as DSED) and inhibited (now RAD) subtypes of RAD. Based on this, the original version of the DAI still aligns with the current conceptualisation of attachment disorder symptomatology.

Each item of the DAI was coded as 0 if symptoms were not present, 1 if there was moderate evidence of symptoms, and 2 if there was strong evidence. Scores are summed creating a symptom score for each disorder, with higher scores indicative of higher severity of Attachment Disorder symptoms. RAD (inhibited) symptoms are assessed by five questions and has a maximum score of ten. DSED (disinhibited) symptoms are assessed by four questions with a maximum score of eight. Zeanah et al., (2002) recommends a cut-off of 3 or more indicating clinical levels of difficulty for each of the subscales. The DAI has been shown to have strong reliability and validity [31].

### Emotional and behavioural difficulties

The Strengths and Difficulties Questionnaire was used to measure emotional and behavioural difficulties (SDQ) [32]. This short carer-report questionnaire measures five domains: emotional symptoms, conduct problems, hyperactivity and inattention, peer relationship difficulties and prosocial behaviours. Items are scored on a three-point scale (0–2); not true, somewhat true and certainly true. Some items require reverse coding. Total difficulties score was the outcome variable used in this study and was calculated by summing the scores for all subscales, apart from prosocial behaviours. This is a screening measure, whereby higher scores are indicative of more difficulties. Goodman, [32] recommends a score of 17 or above potentially indicating clinical levels of difficulty. This measure is commonly used in research and clinical practice and has been demonstrated to have good validity and reliability in children aged 2 to 17 years of age [32]. As the SDQ is only validated in this age group, children younger than 2 years were not administered this measure and thus were not included in the analyses that used this outcome measure (SDQ *N* = 155 at T1).

### Analysis

Statistical analysis was carried out using R Studio (version 4.4.2) [33]. Descriptive statistics were used to characterize types, rates and severity of ACEs in the sample, as well as deprivation levels and age at entry to care. Additional screening of the data was carried out to assess normality and linearity using histograms and scatterplots, respectively. Correlational analyses were conducted using bivariate Pearson correlations among variables across the two time points. Multiple regression was used to explore if ACE cumulative score and maltreatment severity predicted emotional, behavioural and attachment disorder symptoms at T3, above and beyond initial difficulties, whilst also controlling for age at entry to care and deprivation levels. Two models, one for ACE cumulative score and another for maltreatment severity, were estimated for each of the outcomes (SDQ total emotional and behavioural difficulties, RAD symptoms, and DSED symptoms at T3). Treatment arm allocation (from the overarching study) was also controlled for. Only complete data were used in the regression analyses (i.e., missing data were handled with listwise deletion).

A sample size analysis was completed based on previous effect sizes taken from the relevant research. With an effect size of 0.2 for an individual predictor in a multiple regression with 5 predictors, using a power of 0.9 and an alpha level of 0.05, it was estimated we would need a sample of 132 participants. As such, our sample was considered well powered to conduct the analysis.

## Results

### Descriptives and correlations

Around two thirds of the sample had experienced 4 or more ACEs prior to entering care, as assessed by the ACE-Q (M = 4.49, SD = 2.87). Regarding abuse experiences, known exposure to sexual abuse was low in the sample (1.8%), whereas 17.6% of the sample were known to have been exposed to physical abuse, and almost half of the children in the study experienced emotional abuse (45.8%; See Table 1 for breakdown of rates of exposures). Rates of parental seperation and emotional and/or physical neglect ranged between 59.4 and 62.1. Other ACEs were also high in this sample; a third of children had a member of their household in prison, over half had a family member with significant mental illness and over 60% had a family member with substance abuse problems. Over 50% of children were known to have been exposed to domestic violence. Table 2 presents descriptive statistics for outcome measures of the study.

**Table 1:** Adversity Experiences

Adversity Variable	*n*	M (SD)	Observed Range
MCS Maltreatment Total Severity (Abuse and Neglect)	332	10.41 (7.32)	0–36
MCS Abuse Severity	332	2.38 (2.15)	0–11
MCS Neglect Severity	332	8.03 (6.52)	0–31
ACE-Q Cumulative Score	330	4.49 (2.87)	0–9
Type of Adversity Experienced (using ACE-Q)			%
Emotional Abuse			45.8
Physical Abuse			17.6
Sexual abuse			1.8
Emotional Neglect			62.1
Physical Neglect			59.4
Parental Separation			59.4
Domestic Violence			53.9
Substance Abuse in the Home			61.5
Household Member Mental Illness			54.5
Household Member Incarcerated			33.3
Cumulative ACE Scores (using ACE-Q)			%
0			20.30
0–1 “Low”			22.73
2–3 “Medium”			8.79
4+ “High”			68.48

**Table 2:** Descriptive statistics for symptom outcome measure scores

Outcome Measure	*N*	Mean (SD)	Observed Range
SDQ Total Difficulties T1	155	12.53 (8.01)	0–37
SDQ Total Difficulties T3	286	11.61 (7.30)	0–31
DSED Symptoms T1	208	2.49 (2.28)	0–8
DSED Symptoms T3	205	1.37 (1.85)	0–8
RAD Symptoms T1	211	1.34 (1.62)	0–6
RAD Symptoms T3	205	0.69 (1.22)	0–9

The correlations among variables are presented in Table 3. There was a small to moderate and significant relationship between SDQ total difficulties at T1 and T3 (*r* =.29, *p* <.05). SDQ difficulties at T1 were also significantly related to both T1 and T3 RAD (*r* =.47 and *r* =.31, *ps* < 0.05, respectively) and DSED scores (*r* =.35 and *r* =.30, *ps* < 0.05 respectively). For correlations between SDQ total difficulties at T3 and attachment symptoms, there was only significant correlations with RAD (*r* =.48, *p* <.05) and DSED (*r* =.47, *p* <.05) scores at T3, but not at T1.

Cross-sectional correlations between RAD and DSED scores at T1 and at T3 were moderate and significant (all *ps* < 0.05). However, there were no significant longitudinal associations between those two domains.

There were significant positive moderate/strong correlations between age at entry to care and cumulative/total ACE score (*r* =.61, *p* <.05) and maltreatment severity (*r* =.58, *p* <.05). Age at entry to care was also significantly correlated with SDQ and DSED scores at T3, with small correlations (*ps* < 0.05), and did not have any other significant correlations with the remaining outcomes. Cumulative ACE score and maltreatment severity were significantly and strongly correlated with each other (*r* =.69, *p* <.05), but did not have any significant correlations with most outcomes. The one exception was that total ACE score was significantly weakly associated with SDQ total difficulties at T3 (*r* =.22, *p* <.05), and DSED symptom scores at T3 (*r* =.16, *p* <.05). Level of deprivation was not significantly correlated to any of the other variables in this analysis.

**Table 3:** Correlation Matrix (n between brackets)

Variable Name	1.	2.	3.	4.	5.	6.	7.	8.	9.	10.
1. Age at entry to foster care (T1)	1.00									
2. SIMD Decile	0.02 (375)	1.00 (375)								
3. Total ACE score	0.61* (330)	0.02 (329)	1.00							
4. Maltreatment total severity	0.58* (332)	−0.01 (331)	0.69* (330)	1.00						
5. SDQ total difficulties T1	−0.01 (155)	−0.12 (153)	0.09 (138)	0.08 (138)	1.00					
6. SDQ total difficulties T3	0.12* (286)	0.03 (284)	0.22* (262)	0.11 (264)	0.29* (125)	1.00				
7. DAI RAD score T1	0.04 (211)	−0.10 (209)	−0.10 (189)	0.00 (189)	0.47* (138)	0.06 (180)	1.00			
8. DAI RAD score T3	0.12 (205)	−0.02 (203)	0.14 (188)	0.02 (190)	0.31* (92)	0.48* (205)	0.15 (129)	1.00		
9. DAI DSED score T1	−0.09 (208)	−0.11 (206)	0.04 (187)	0.00 (187)	0.35* (136)	0.10 (178)	0.52* (208)	0.17 (127)	1.00	
10. DAI DSED score T3	0.16* (205)	0.04 (203)	0.16* (188)	0.10 (190)	0.30* (92)	0.47* (205)	0.11 (129)	0.64* (204)	0.27* (127)	1.00

*p<.05

### Multiple regression analysis

Tables 4 and 5 include the results from the regression analyses used to explore predictors of symptoms at follow up (T3) for total emotional and behavioural difficulties, RAD and DSED symptoms, respectively. Predictors of interest were primarily ACE-Q cumulative total score, maltreatment severity, and age at entry to care, while controlling for outcome score at T1, deprivation, and treatment arm^1^. None of the models demonstrated issues with multicollinearity based on the variance inflation factors assessed.

Age at entry to care and severity of maltreatment score did not uniquely predict any of the outcomes at Time 3. Level of deprivation and treatment arm also were not significant covariates in any of the models. ACE score in the emotional and behavioural difficulties model, significantly predicted SDQ total difficulties score at Time 3, even when controlling for other predictors (Table 5; β = 0.21, *p* =.03). Total ACE scores did not predict attachment disorder symptoms over time.

**Table 4:** Multiple Regression Analysis of Emotional and Behavioural Difficulties

Outcome: SDQ total difficulties at T3				
Model 1: ACE-Q	b	β	t	p
Age at T1 (age at entry to foster care)	−0.17	−0.02	−0.27	0.79
SIMD Decile	−0.30	−0.04	−0.48	0.63
Total ACE score	0.86	0.21*	2.27	0.03
SDQ total difficulties at T1	0.21	0.23*	2.49	0.01
Treatment Group	−1.43	−0.09	−1.06	0.29
Model F-statistic (DF = 5, 109) = 2.86, *p* =.0182, Multiple R-squared = 0.12				
Model 2: MCS Maltreatment Severity				
Age at T1 (age at entry to foster care)	−0.32	−0.05	−0.51	0.61
SIMD Decile	−0.12	−0.02	−0.18	0.86
Total Maltreatment Severity	0.19	0.15	1.56	0.12
SDQ total difficulties at T1	0.22	0.25**	2.65	< 0.01
Treatment Group	−1.23	−0.08	−0.90	0.37
Model F-statistic (DF = 5, 109) = 2.28, *p* =.0521, Multiple R-squared = 0.09				

*p<.05

**p<.01

**Table 5:** Multiple Regression Analysis of Attachment Disorder Symptoms

**Outcome: Symptoms of RAD at T3**				
Model 1: ACE-Q	b	β	t	p
Age at T1 (age at entry to foster care)	−0.05	−0.05	−0.49	0.63
SIMD Decile	−0.10	−0.08	−0.87	0.39
Total ACE score	0.02	0.03	0.28	0.78
RAD at T1	0.08	0.11	1.14	0.26
Treatment Group	−0.03	−0.01	−0.15	0.88
Model F-statistic (DF = 5, 112) = 0.56, *p* =.730, Multiple R-squared = 0.02				
Model 2: MCS Maltreatment Severity				
Age at T1 (age at entry to foster care)	−0.02	−0.02	−0.26	0.79
SIMD Decile	−0.10	−0.08	−0.86	0.39
Total Maltreatment Severity	−0.02	−0.11	−1.12	0.27
RAD at T1	0.07	0.10	1.06	0.29
Treatment Group	−0.04	−0.02	−0.19	0.85
Model F-statistic (DF = 5, 112) = 0.80, *p* =.551, Multiple R-squared = 0.03				
**Outcome: Symptoms of DSED at T3**				
Model 1: ACE-Q				
Age at T1 (age at entry to foster care)	0.002	0.001	0.01	0.99
SIMD Decile	−0.03	−0.02	−0.16	0.87
Total ACE score	0.11	0.10	1.05	0.30
DSED at T1	0.17	0.22*	2.36	0.02
Treatment Group	0.04	0.01	0.11	0.91
Model F-statistic (DF = 5, 110) = 1.35, *p* =.25, Multiple R-squared = 0.06				
Model 2: MCS Maltreatment Severity				
Age at T1 (age at entry to foster care)	0.01	0.01	0.07	0.95
SIMD Decile	−0.01	−0.005	−0.05	0.96
Total Maltreatment Severity	0.01	0.03	0.35	0.73
DSED at T1	0.16	0.22*	2.34	0.02
Treatment Group	0.05	0.01	0.15	0.88
Model F-statistic (DF = 5, 110) = 1.15, *p* =.341, Multiple R-squared = 0.05				

*p<.05

**p<.01

#### Post Hoc analysis

There were no significant predictors of attachment disorder symptomatology (RAD or DSED) at T3, based on the variables explored in this analysis, and only DSED showed some level of stability over time (β = 0.22). As such further exploratory, post hoc analysis was carried out to examine how these symptoms changed over time. Histograms for both RAD and DSED change scores were visualised with no concerns with normality noted. Paired T-tests were run to compare RAD and DSED symptom scores between T1 and T3. This demonstrated that both RAD and DSED symptoms decreased over time, as scores were significantly lower at T3 than at T1 (t = −3.85, *p* = < 0.001 and t = −3.72, *p* = < 0.001 respectively). 

## Discussion

This study explored emotional, behavioural and attachment disorder symptoms in a large sample of care-experienced young children over a period of two and a half years in care, and their associations with age at entry to care and adversity histories. Strong associations between age at entry to care and history of adversity were found, suggesting that young children entering care at older ages were at risk of having experienced more ACEs and higher severity of maltreatment. In exploring the effect of adversity on symptoms over time, cumulative ACE score predicted higher emotional and behavioural difficulties in children following two and a half years in care. In contrast, none of the predictors were associated with attachment disorder symptoms at two and a half years in care, which were significantly lower as compared to initial levels, offering promising findings for young children in care.

### Adversity histories

This study was able to characterise the adversity history of a Scottish sample of very young children in foster care. Rates of known sexual and physical abuse in this sample were relatively low when compared to rates reported with older care-experienced children [34], and yet in line with previous research with young children in care [6]. Compared to general populations of children, care-experienced young children in this sample were disproportionately affected; research from the National Society for the Prevention of Cruelty to Children (NSPCC) in the UK reports rates of 0.1% and 1.3% for sexual and physical abuse, respectively, in their ‘under 11 years old’ nationally representative sample [35].

Rates of recorded emotional abuse and neglect in these young children were very high, with emotional neglect being the most experienced adversity in this sample, at 62%. These rates were similar to prevalences shown in whole childhood care-experienced samples and again, significantly disproportionate to non-care experienced populations [34, 35]. Wider adversities were also prevalent in the sample, with large proportions of the children having been exposed to domestic violence, and parental substance misuse, incarceration, and mental health difficulties. Overall, our characterisation of this sample shows the high levels of adversity experienced in care-experienced young children and despite their young age, they had experienced similar cumulative ACE totals as care-experienced older children in Scotland (Gibson, 2020). Around two thirds of participants had experienced four or more ACEs; a figure repeatedly found to be linked to significant psychological, social and physical health difficulties in later life [25]. In this respect, this young population requires multisectoral attention and intervention to prevent the occurrence of ACEs in the first instance and mitigate their negative sequelae when they do occur.

### Effect of adversity on attachment disorder, emotional and behavioural symptoms

Our longitudinal findings show that cumulative ACEs score, based on exposure prior to entry to care, predicted an increase in emotional and behavioural difficulties over a 2.5 year period in care. That is, the more adversity types a child experienced before entering care, the higher their difficulties would be two and a half years later, even when controlling for initial levels of emotional and behavioural difficulties. These findings are consistent with the cumulative risk model and literature demonstrating the chronic and long-term impact of experiencing multiple types of adversity [3, 25]. This finding suggests that the removal into appropriate and supportive foster care placements, by itself, does not fully protect against the long-term impact of adversity on emotional and behavioural difficulties and targeted identification and prevention of early adversity in the first place remains paramount.

Notably, the same effect was not shown for maltreatment severity, despite there being a significant relationship between ACE total scores and maltreatment severity scores. There is large body of literature surrounding how to best conceptualise and measure trauma and adversity, and studies differ based on how adversity is operationalised such as by adversity type(s), deprivation versus threat, exposure frequency or severity [36, 37]. It may be that operationalising impact through severity is not as relevant in predicting later difficulties as cumulative total in this sample. Perhaps severity measurement introduces more subjective rater judgements that might not fully capture the experience of a young child, or that cumulative scoring more accurately represents the complexity of ACE co-occurrence. There could also be developmental differences in terms of when perceptions of severity are more strongly related to outcomes, which can be explored in future research looking at the complex dimensions of childhood adversity.

When exploring attachment disorder symptomatology, we found a small correlation between ACE total score and DSED symptoms at follow up, consistent with associations reported in older populations [38]. Nonetheless, when we explored the unique effects from cumulative ACE total or maltreatment severity on attachment disorder symptoms over time, while controlling for initial levels and other factors like age at entry to care, we did not find any effects of adversity on attachments disorder symptoms. This is surprising considering DSED and RAD are the only two disorders *specifically* linked to childhood maltreatment [11]. When exploring this further, it was found that symptoms of both attachment disorders did not show strong stability overtime, and there were reductions in both DSED and RAD over the 2.5 years in care. This is a clinically positive finding, demonstrating that for young children entering care after adversity, attachment disorder symptoms may improve when placed in appropriate care settings and is in line with findings with children placed in adoptive families [9]. This also aligns with findings from the Bucharest Early Intervention Project, where early foster care placement resulted in steeper reductions in RAD symptoms over time versus institutional care [39]. While this study did not explore potential mechanisms for this reduction, research in the area has highlighted possible contributors such as higher quality caregiving resulting in attachment security and stability, and reduced exposure to further ACEs [40, 41]. These opportunities arising from entering foster care may buffer some of the negative impacts of early experiences.

When taken in combination with our other findings, however, it is still important to highlight the stability in emotional and behavioural difficulties over time, and the increase in those difficulties as a function of pre-care ACEs. Therefore, even though attachment disorder symptoms appear to reduce when children are removed from an unsafe environment, their emotional and behavioural needs may still require additional supports, including specialist mental health assessment, monitoring over time, and intervention.

### Age at entry to care

While age at entry to care did not uniquely predict mental health outcomes at 2.5 years in care, it was significantly and strongly associated with pre-care cumulative ACE score and maltreatment severity. These results suggest that young children who were placed in care at an older age were exposed to more ACEs and more severe maltreatment prior to entry to care. This effect is notable considering the very young age of this sample and considering the impacts of ACEs on emotional and behavioural outcomes well into children’s care journey. Even with relatively early social work intervention (under five years of age), these children were still vulnerable to experiencing extremely high levels of adversity. Again, this demonstrates the importance of early identification of risk and implementation of support for highly vulnerable families to ensure the future wellbeing of young children.

Our correlational analysis also demonstrated a small but significant relationship between age at entry to care and emotional and behavioural difficulties and DSED symptoms at T3. These findings are in line with previous, initial research in this area by Hillen and Gafson [21], who published a cross-sectional study with a small sample of young children in London. They found a significant association between entering care after 6 months of age and having a mental health difficulty (including behavioural, emotional and/or attachment disorders). These cross-sectional and longitudinal associations are noteworthy, especially in a young sample of children whose outcomes may not show until later in development.

The regression analysis finding that age at entry to care did not uniquely predict measured outcomes at T3, when adversity and baseline symptoms were controlled for should not be taken to mean that age at entry to care or time spent in adverse settings is not associated with later outcomes. We were looking to see whether age at entry to care had an effect above and beyond the effect of adversity and baseline symptoms, rather than whether on its own is predictive of later outcomes. As we know, other work that has examined extreme forms of early deprivation, such as in the longitudinal English and Romanian Adoptees (ERA) study found robust effects of age at adoption (older than 6 months of age, versus younger) on outcomes persisting into adolescence and beyond [19]. Our correlational results are consistent with these findings and suggest that time spent in adverse settings is associated, albeit weakly, with negative outcomes at later time points. The lack of unique predictive effects of age at entry to care in our model may be due to several reasons. It is possible that entering it alongside adversity and maltreatment scores that its effects were diminished due to their associations. In addition, it is possible that the restricted age range in our current study did not allow us to detect these effects and that further follow ups into later childhood and adolescence may offer opportunity to examine this effect on outcomes that become apparent later in childhood and adolescence, such as emotional difficulties.

### Strengths, limitations and further research

In this important investigation of outcomes of young care-experienced children, we utilised longitudinal data from a large sample of care-experienced young children with detailed history of adversity and maltreatment severity and validated measures of attachment disorders and emotional and behavioural difficulties. Additionally, the larger trial overall had a high recruitment and retention rate [23]. Nonetheless, there are limitations of note. Firstly, the majority of participants lived within the lowest SIMD areas, and none lived in the three least deprived areas. There was perhaps not enough variation in the sample to see the impact of deprivation on outcomes, as the influence of this has been well established in previous literature [42]. Second, the design of the larger trial allowed for all eligible families in a large geographical area to be offered participation and had a recruitment rate of about 60%. However, it is not possible to know if the families that declined to take part were characteristically different, and therefore how representative or generalisable the results are. Third, an expected limitation of longitudinal designs is attrition, as shown by the missingness of variables across timepoints. Nonetheless, the numbers present offered a well powered study, and there were no significant differences between those who completed the trial and those who dropped out early [23].

The measure used to assess emotional and behavioural difficulties in this study was the SDQ [32]. While this measure is well used in the literature, it is only standardised in children aged two or above. Therefore, we were unable to characterise the emotional and behavioural difficulties of very young infants, leading to a proportion of the sample not being included in models with the SDQ outcome. Further, in our regression models we used only complete data which results in data loss and potentially introduces bias to the results. In addition, although linear regression is relatively robust to deviations in the normality assumption [43], it is important to note distributional issues with outcome variables and consider alternative approaches in the future. Furthermore, there are other factors that could be important covariates or moderating factors that were not measured or included in these analyses. This includes placement instability and the quality of relationships while in care. Finally, further longitudinal research would be helpful to assess if the established predictors have longer lasting effects beyond the 2.5 years explored in the current study.

## Conclusion

Our findings demonstrate that, in a sample of young children in foster care, most had experienced a high number of ACEs including maltreatment and other types of adversity such as domestic violence and parental substance misuse. Those children were more likely to have been exposed to more ACEs and severe maltreatment the later in age they entered care. It was found that cumulative adversity predicted an increase in emotional and behavioural difficulties after 2.5 years in care, highlighting the need for further support and monitoring for this population. Regarding attachment disorders, average symptoms of RAD and DSED were lower at 2.5 years in care than initial levels. This may demonstrate that placing young children in supportive and safe environments can help reduce some of their symptoms, but that additional psychological supports may still be required for the emotional and behavioural wellbeing of those children. Importantly, the results speak to the continued need for early prevention of ACEs, and support for families to provide safe and stable environments for young children.

## Data Availability

Data requests will be processed by the Trial Management Group (TMG), taking into consideration the scientific rationale, ethics, logistics and resource implications of the requests. Data access requests should be submitted by email to the chief investigator on the grant (H.M). If requests are approved, a collaboration agreement would be expected. Consideration of any cost implications, and cost recovery would be expected on a not-for-profit basis.

## Footnotes

1 Models were run with an interaction effect between predictor and treatment arm assignment to investigate whether treatment arm moderated any of the results. However, none of the interaction terms were significant (*p* >.05), and therefore the analysis only retained treatment arm as a covariate in the model.
